# Supplementary material for: Artificial accommodating intraocular lens powered by an ion polymer-metal composite actuator
Source: PLoS One. 2021 Jun 23;16(6):e0252986. doi: 10.1371/journal.pone.0252986 (PMC8221497; doi:10.1371/journal.pone.0252986)
Supplement: Attachment — Submitted filename: MyAnswer.docx [file pone.0252986.s002.docx]

This is Tetsuya Horiuchi. Thank you for your suggestion.

Black letters sentences are your comments.

Purple letters sentences are my answers.

Red letters sentences are sentence which is written in my paper.

(2) The lens design is based on the authors’ previous study [Horiuchi et al., Opt Express 24 (2016) 23280; REF 4 in the manuscript]. However, it is unclear and problematic why the authors did not cite their other work that is more closely related [Horiuchi et al., Smart Mater. Struct. 26 (2017) 045021].

I added my paper (2017) in reference.

(line 129-130)

In the previous study, we conducted experiments using the swine lenses as it was very difficult to make a lens soft and clear enough for use in optical experiments [15].

(3) Based on their earlier works, using IPMC, system design are not new. Use of so-called ‘gel lens’ (instead of swine lens) seems to be a new idea from the authors’ perspective. This is a major material change and is novel. At the same time, information about this material must be provided properly – and this is an issue. Other claimed novelty includes the elimination of sixth-order aberration by using the material (although this term was not properly defined in the manuscript.)

Sorry, and I agree my sentence gave an erroneous impression. Our novel idea is not using gel with IPMC actuator but explained in Fig. 5 and chapter "Power supply". It is actually new, and recently we already get patent for this. Of course, there are a lot of problems for moving this idea into actual surgery (as you commented later). This paper is pilot study for this.

Make it more clearly, I added sentence in chapter “Previous work”, and in here I used your suggested two references.

(line 77-81)

Our aim was to develop a new control system for changing the focus of an accommodating IOL with a polymer actuator. The concept of a lens control system with a polymer actuator is not novel. Carpi et al. [12] and Maffi et al. [13] suggested a tunable silicone lens, which is driven by dielectric elastomer actuators. Moreover, Kikuchi et al. [14] suggested a variable-focal length lens, which is controlled by an ion polymer metal composite (IPMC) actuator. However, their aim was to create a lens for camera-like devices, not for accommodating an IOL.

(4) Generally speaking, this manuscript lacks critical information in many important fields so that the manuscript leaves many unanswered questions that hinder its ability to argue its significance and novelty. Writing itself have many issues to be considered for academic publication.

I’m very sorry, especially I did not write the properties of silicone and image of sixth order aberration. I added some experiments, figures, and chapter. For later comments, I will explain detail.

DATA ANALYSIS SUPPORTING THE CLAIMS

(5) Related with point (3), the authors must provide critical material information related with their so-called ‘gel lens’. What is the transparency vs wavelength? (UV-vis spectra must have been provided at the least) What is the mechanical property of the silicone KE-109 A mixed with KF-96-20-CS that was mixed at 1:1:8 ratio? (Rheological measurement information must have been provided) Did it have a ‘jacket’ layer to contain any unreacted silicone from oozing out? What was the refractive index of the ‘gel lens’, as a function of wavelength? Was it, at least, consistent? Whereas the ‘gel lens’ is the key for the novelty of this manuscript, there are no information about these in this manuscript.

I have two answers. First is about properties of the silicone, and the latter is about “jacket” layer.

I added chapter “Properties of the silicone”. And I measured transmissivity, refractive index. But we have no tools to measure Rheological measurement information. So, as substitute for this, we measure Young's modulus.

(line 151-172)

“Properties of the silicone”

We measured three properties of the silicone, i.e., transmissivity, refractive index, and Young's modulus.

Transmissivity is shown in Fig. 8. We prepared the cuboid-shape silicone with a thickness of 3.5 mm. We placed it in air, applied 250 nm to 1000 nm wavelength light, and calculated the transmissivity of the silicone. In the visible-light area, transmissivity was higher than 85%, and considering that the Fresnel reflection is approximately 3%, it was actually almost 90%.

Fig. 8. Transmissivity of silicone. This is the Fig. 8 legend.

The refractive index is shown in Fig. 9. We used an industrial microscope (USPM-RU-W, Olympus, Tokyo) and a ×10 objective lens (NA=0.12) to measure the Fresnel reflection from a small surface area of the lens material. As the confocal microscope can measure the reflection of very small area, the measurement is not affected by the surface condition. We selected a flat and clean area to avoid including unwanted reflection components other than the Fresnel component. We repeated the measurements three times. Refractive indices were calculated from the measured reflection rates using the Fresnel reflection law. In the area of the optical wavelength, the refractive index was 1.40–1.41, which is a quite common value for the refractive index of silicone.

Fig. 9. Refractive index of silicone. This is the Fig. 9 legend.

The Young's modulus of our silicone was 57.0 kPa. Considering that the Young's modulus of KE-109 silicone is 950 kPa, our silicone was more than 16 times softer than the original one. Actually, the rheologic properties are also important when considering such a very soft material. However, our IOL system does not inflict large deformations to the lens, i.e., there is less than 0.1-mm deformation to a 6-mm thickness lens; therefore, we did not consider this.

And about “jacket” layer. In this paper, we did not discuss. But I agreed this problem is very important with the object of biological compatibility. And I added new chapter.

(line 232-238)

“Biological compatibility”

This was a pilot study, and, therefore, we did not select lens material with the objective of biological compatibility. However, to consider the surgical applications of this technology, we should discuss the coated layer. This coated layer requires high biological compatibility, has to be very soft and sufficiently thin to avoid preventing the movement of the IPMC actuator and lens, and transparent to allow light to pass. We watchfully await the results of the Fujie group’s study [18] regarding this coated layer; they could create a membrane thinner than 1 μm, which would be biologically compatible. In the future, we should test an accommodating IOL system that would be fully covered by this film.

(6) Lines 63-65 sum up the main problem according to the authors: “The conventional accommodating IOL can be used to control the focus of the lens with the patient’s ciliary muscle; however, a significant problem exists. The accommodating ranges vary widely from patient to patient. This phenomenon cannot be resolved by these methods while the patients’ ciliary muscles are used for accommodating”. Two questions I have are:

a) There is no source provided for this. How large is the variance in accommodating range among patients? i.e. how many patients struggle with using accommodating IOLs due to lack of ciliary muscle function?

My co-author, Dr. Mihashi is past time, a member of the group to study Tetraflrx accommodating IOL. And he wrote paper of this.

(URL is <https://journals.healio.com/doi/10.3928/1081597X-20100114-04>).

Because of concerned interests, he cannot open detail information, but only he can say it is far from “controlled”. By this experience, he thought it is too difficult to connect “Biological” muscle with “Artificial” lens, and he desired “Full-artificial” accommodating IOL. For this muscle, he found my lab’s IPMC actuator, and we start this study in 2015.

And next is, ”how many patients struggle with using accommodating IOLs due to lack of ciliary muscle function?”. Main factor for losing accommodating power is lens hardening. Of course, ciliary muscle is weakened by age as the other muscle. But it is not main factor.

b) Etiology of presbyopia is actually disputed [DeBoer et al., IEEE Transactions on Biomed. Eng., 64 (6), 1129-1135 (2016); REF 14 in the manuscript] and one theory suggests that presbyopia occurs primarily due to hardening of the lens itself. Conceivably, in some cases patients retain most ciliary muscle function at the time of cataract surgery. Is this type of actuator-based IOL still beneficial or even viable for patients of this nature?

My answer is "YES", our IOL is workful. Our accommodating IOL is set with lens. Of course, white-outed and harden lens is removed by surgery, and after that, our accommodating IOL is inserted.

(7) Although it arguably led to the most significant improvement in results (elimination of sixth-order aberrations) the design features and properties of the “hard ring” are never fully examined and discussed. Is the composition of this material significant? For example, “Then, we attached the hard ring and pressed it such that the shape of the inside of the ring was curved like a lens” (lines 143-144) is quite difficult to visualize. Closer examination of the part geometry around the ring (and the ring itself) would be helpful (maybe in Figure 3, 6, or 7), especially since changes to the shape of the ring are suggested as a potential improvement to prevent noise.

I agree this part is very important. I added sentence and new figure 14.

(line 210-216)

In Fig. 14, we illustrate the sixth order aberration result. The sixth order terms of the Zernike polynomials are parameters that parameters that estimate the degree of sixth order aberration presence. In this study, the azimuth sixth order terms were very meaningful because they were found to be large in previous studies. This is because, previously, the lens was deformed by a six-winged actuator.

Additionally, by comparing the calculation results, we found that we could reduce Zernike 6. There was a 0.30-μm over 4-mm-radius circle area in our previous study, while it was 0.10 μm in our current study.

(8) It is suggested that a potential improvement is switching to a liquid balloon lens. However, earlier in the article it was mentioned that liquid crystal lenses were unsafe to use at voltages >1.5 V due to electrical leakage. This suggests that the experimental conditions used in this manuscript (voltages up to 2.25 V) would not be feasible if the switch to a liquid lens was made.

Sorry, but I think you may misunderstand liquid balloon lens and liquid crystal lens. Liquid balloon lens is liquid covered by clear lens shape jacket. The softer lens becomes, the smaller voltage is needed to deform.

(9) What is the precedent for embedding a stable and non-degrading power source, complete with added logic circuit, in the eye sockets? It seems that such a design would be prone to eventual failure with so much movement from the eyeball occurring in the area. Is it also possible that this will prohibitively increase the complexity and complications of the cataract surgery?

I have two answers.

First, I don't put power source and logic circuit in eye socket. The advantage of my idea is I can put such a thing out of the eyes.

However, it is answer for second question. Even if I don't put such a thing in eye socket, cataract surgery becomes very complicate and difficult. This have to be solved by future work.

(10) The IPMC that the authors used: What is the time required to adjust the focal length? Is the IPMC reaction time comparable to what our eyes can do?

It is very difficult question. First, I want to answer IPMC reaction time. Our current system, it is within 1 second. But I have to consider when we can make good lens system which can control larger accommodating range, it means also it takes more time to control. It is future works problem.

And nest is “What is the time required to adjust the focal length”. For this problem, I and my co-authors have different opinion each other. One opinion is “It is required within one or two seconds because young healthy people can do in this time”, and the other opinion is “It is not required and even one minutes is no problem, because the accommodating power of over 60 years people is almost zero. So, only IOL with accommodating skill exist, first step is clear.”. I cannot say which is correct and what is required time.

WRITING ISSUES

(10) In addition to the lack of the necessary details, the literary value of the manuscript itself must improve before being considered for publication. Introduction and Conclusion must flesh out better. The results and discussion parts needs reorganization after including the necessary details provided above.

This paper is emended by Editage company, and I think quality is enough.

REFERENCING ISSUES

(11) This manuscript does not give fair treatment for other group’s recent works. For example, a recent review paper [Mylona and Tsinopoulos, Pharmaceauticals 13 (2020) 448 and 80 references therein] includes recent advances in the field of IOL. There have been many exciting studies on silicone-based lens with artificial muscles and material analysis, for example [Maffi et al., Adv Funct Mater 25 (2015) 1614; Carpi et al., Adv Funct Mater 21 (2011) 4152].

Thank you. [Maffi et al., Adv Funct Mater 25 (2015) 1614] and [Carpi et al., Adv Funct Mater 21 (2011) 4152] are nice paper to explain my study. I added these into reference.

(line 77-79)

The concept of a lens control system with a polymer actuator is not novel. Carpi et al. [12] and Maffi et al. [13] suggested a tunable silicone lens, which is driven by dielectric elastomer actuators.

But I am very sorry to say I cannot add review paper [Mylona and Tsinopoulos, Pharmaceauticals 13 (2020) 448 and 80 references therein]. I read this carefully, and I understand this paper aimed drug and cells. My paper focuses mechanical problems. And I think it is difficult to connect my paper with this review paper. Substitute for this, we use another paper Kikuchi et al. They are studying variable-focal length lens with IPMC actuator.

(line79-80)

Moreover, Kikuchi et al. [14] suggested a variable-focal length lens, which is controlled by an ion polymer metal composite (IPMC) actuator.

MINOR ERRORS

(12) Small typo (also present in previous paper by this author), “NuLans” should be NuLens [Alio et al., J. Cataract and Refractive Surgery, 35 (10), 1671-1678 (2008)].

I corrected.

(13) Refs [5] and [6] citation sequence?

I corrected.

(14) “hydatoid circulation”  explanation needed for general readers

I corrected.

(15) “Usually, silicone is made by mixing the two liquids under 100oC … too hard to use as an accommodating IOL.”  I believe that the authors refer to Sylgard 184. There are many silicones – just going to local hardware store can assure that there are many soft silicones curing at room temperature.

Sorry, my sentence gave an erroneous impression to you. "Too hard to" in this sentence is not mean "too difficult to", but I "too rigid to". I change sentence.

(line 143-144)

This silicone is made by mixing the two liquids under 100℃ heat for longer than 1 hour. However, the usual type is too rigid to use as an accommodating IOL.
