# Supplementary material for: Artificial accommodating intraocular lens powered by an ion polymer-metal composite actuator
Source: PLoS One. 2021 Jun 23;16(6):e0252986. doi: 10.1371/journal.pone.0252986 (PMC8221497; doi:10.1371/journal.pone.0252986)
Supplement: Attachment — Submitted filename: Response to Reviewers 20210421.docx [file pone.0252986.s003.docx]

2021/04/21

Reviewer comments were addressed. More supporting data could have been revealed (for example, where are the evidences for the transmittance 'almost 90%'?)

My Answer

Thank you for your comment. I attached one file in “Supporting data”. This is raw data file to calculate refractive index. We calculated refractive index by measuring spectral reflection factor. This is 2.5-3.0%. So, we said actual transmissivity was almost 90%.
